# Artificial Intelligence-Based Voice Assessment of Patients with Parkinson’s Disease Off and On Treatment: Machine vs. Deep-Learning Comparison

**DOI:** 10.3390/s23042293

**Published:** 2023-02-18

**Authors:** Giovanni Costantini, Valerio Cesarini, Pietro Di Leo, Federica Amato, Antonio Suppa, Francesco Asci, Antonio Pisani, Alessandra Calculli, Giovanni Saggio

**Affiliations:** 1 Department of Electronic Engineering, University of Rome Tor Vergata, 00133 Rome, Italy; 2 Department of Control and Computer Engineering, Polytechnic University of Turin, 10129 Turin, Italy; 3 Department of Human Neurosciences, Sapienza University of Rome, 00185 Rome, Italy; 4 IRCCS Neuromed Institute, 86077 Pozzilli, Italy; 5 Department of Brain and Behavioral Sciences, University of Pavia, 27100 Pavia, Italy; 6 IRCCS Mondino Foundation, 27100 Pavia, Italy

**Keywords:** speech, voice, Parkinson’s disease, artificial intelligence, deep learning, CNN, SVM, L-Dopa, F0

## Abstract

Parkinson’s Disease (PD) is one of the most common non-curable neurodegenerative diseases. Diagnosis is achieved clinically on the basis of different symptoms with considerable delays from the onset of neurodegenerative processes in the central nervous system. In this study, we investigated early and full-blown PD patients based on the analysis of their voice characteristics with the aid of the most commonly employed machine learning (ML) techniques. A custom dataset was made with hi-fi quality recordings of vocal tasks gathered from Italian healthy control subjects and PD patients, divided into early diagnosed, off-medication patients on the one hand, and mid-advanced patients treated with L-Dopa on the other. Following the current state-of-the-art, several ML pipelines were compared usingdifferent feature selection and classification algorithms, and deep learning was also explored with a custom CNN architecture. Results show how feature-based ML and deep learning achieve comparable results in terms of classification, with KNN, SVM and naïve Bayes classifiers performing similarly, with a slight edge for KNN. Much more evident is the predominance of CFS as the best feature selector. The selected features act as relevant vocal biomarkers capable of differentiating healthy subjects, early untreated PD patients and mid-advanced L-Dopa treated patients.

## 1. Introduction

Since systems based on artificial intelligence (AI) have become ubiquitous, their capability to support clinical practices in healthcare has been increasing as well. The frontiers of technological progress in this area are constantly expanding and reaching areas previously considered only accessible to human experts [1], notably thanks to the wide diffusion of wearable sensors [2,3] or devices [4] that can be exploited for the collection of physical signals used as a data source for AI algorithms [5].

The worldwide diffusion of 6.5 billion of smartphones (owned by about 83% of the population) [6], with over 11 billion devices connected to the Web including wearables [7], has allowed for health monitoring from anywhere and at any time [8].

Parkinson’s Disease (PD) is a neurodegenerative disorder caused by the progressive degeneration of dopaminergic neurons, which especially occurs in the substantia nigra pars compact region of the midbrain [9]. PD is the second most common neurodegenerative disorder (after Alzheimer’s disease), affecting about 6.1 million individuals in 2016, likely to become 9 million by 2030 [10].

Significantly, PD patients face various motor and non-motor disorders, including walking, sleeping, and speech impairments. In particular, recent evidence demonstrates that speech alterations can arise up to 10 years earlier than cardinal motor impairment manifestation [11], so that their assessment can advantageously lead to early diagnoses. In this context, it is to be taken into consideration that PD diagnosis is usually performed with empirical assessments, linked to the visible presence of symptoms. On the one hand, this calls for methods for identifying prodromal PD signs, and on the other hand, opens up interesting possibilities within the realm of remote healthcare, AI-aided pre-diagnostics and sensor-based analyses.

It is estimated that approximately 75–90% of PD patients present abnormal speech [12], which makes the study of voice production a powerful tool for early identifying, monitoring, and following up of PD, to be added to the assessment of motor symptoms such as bradykinesia, rest tremor, rigidity or postural and gait impairment [13,14]. 

Human voice production occurs through complex and synergistic movements of systems and subsystems (vocal cords, larynx, glottis, oral cavity, and more), which can be affected by the speaker’s health condition [15]. As [16] and others highlight in particular, PD involves dramatic, objective, and measurable changes in voice production, which can include (among others) increased noise levels (due to an incomplete vocal fold closure) and voicing leakage (i.e., altered voiced/unvoiced transitions, due to the difficulty to perform fine start- and stop-movements). While speech impairment assessment can indeed be performed through laryngoscope and video-stroboscopic tools, these are very expensive and time-consuming examinations [17].

Currently, speech impairment is assessed mainly through neurological examinations, alongside questionnaires about the patient’s work, hobbies, and daily routine, to rate different aspects including volume, prosody, and clarity. The Movement Disorder Society Unified Parkinson’s Disease Rating Scale (MDS-UPDRS) is the standardized rater-dependent clinical tool to evaluate the severity of the impairment. This approach is effective, but with some limitations due to the influence of the skill and experience of the rater, and to the effect of the “narrow” scale (scores with integers within 0–4) that can poorly reflect real differences between two equally-scoring patients. Moreover, outpatients’ visits are infrequent (usually a few times a year at most), so potentially influenced by the patient’s specific status at the time of the evaluation procedure (e.g., sleep quality [18], emotional status [19], hour of the day).

Properly validated AI tools can reduce the possible subjectivity bias and “enrich” the scale when applied to the vocal test (even enabling daily evaluations), as different studies have already demonstrated [11,12,20,21,22]. However, the human voice can be potentially influenced by other issues ranging from environmental conditions to subject-specific characteristics [23,24,25], so that other forms of evidence are mandatory. In particular for PD, the effect of medication on speech production is still poorly addressed, with results ranging from no effects [26] to meaningful ones [27], while the differences can even depend on the specific phonemes investigated [23,28,29]. The voice damage experienced by patients with PD is typically characterized as “hypokinetic dysarthria”, the primary issues of which involve articulation and breathing difficulties as well as a voice quality that is empirically described as “trembly” and “unstable”. Although other ML-based methodologies, mainly applied to EEG and MRI, have been proposed for the detection of PD, vocal analysis has demonstrated its effectiveness as a reliable detector, since around 90% of PD patients have dysarthric symptoms [30]. Other than being a reliable means to non-empirically quantify voice impairment in diseases that affect phonatory production, voice analysis is also a completely non-invasive, low-cost and pseudo-real-time solution for deploying telemedicine assessments. Voice-based AI solutions have been successfully experimentally investigated and employed in other medical fields such as dysphonia [31,32,33], COVID-19 and pulmonary diseases [20,22,34,35], and even emotion and stress recognition [24,36]. 

A recent work [37] reports a critical review of pathological voice characterization approaches, evidencing the methodological issues potentially hampering performance assessment trustworthiness, including the database dimension and a stratified corpora (either among classes or genders). Controversy over which specific Artificial Intelligence (AI) approach to employ, namely Machine Learning (ML) vs. Deep Learning (DL), was also considered, which involves the differences in their data-driven and model-driven nature. Although DL is proved to be very effective, the use of low-interpretability models may however evoke the resistance of clinicians asking for high-level evidence in clinical practice, in turn resulting in the overfitting phenomenon as well as a lack of generalization. ML and DL models for the assessment of PD were compared mainly for binary classification tasks (healthy vs. PD), involving acoustic features as an input to ML pipelines: apart from a few exceptions [38,39], the majority of studies reported better performance from of DL models [38,39,40,41,42,43,44,45]. Similar results are also reported in works employing deep features extracted from spectrograms [46,47,48,49,50,51]. However, most published works employ very limited datasets (usually with less than 50 subjects) and/or feature sets, and no comparison has been made between a more comprehensive range of the stages of the disease, nor in the data regarding the medication or the impairment level. Most of the studies are limited to a hold-out validation, splitting the dataset into train and test subsets only once, and only a few performed cross-validation [41,49] which is the de-facto standard in traditional ML studies.

To build a comprehensive baseline for voice analysis for PD detection, we strove to present a thorough literature review [52], especially directed towards feature-based methodologies, which we will refer to for more detailed statistics. For the sake of completeness, a brief overview of relevant works is also presented in Table 1: both ML and DL approaches are covered, with the main limitations often involving small or poorly recorded datasets as well as a general lack of interpretability. To face these challenges, we strove to build an extensive, well-prepared dataset involving a grand total of 426 subjects, without relying on crowdsourced, non-validated data. Other common datasets, as detailed in [52], are those presented by Little, Naranjo and Tsanas that involve a small amount of PD subjects, along with Sakar’s one involving 180 PD subjects recorded in unspecified conditions with unspecified devices, as well as the larger mPower dataset of over 1000 subjects, but the recordings for which are crowdsourced and unverified. 

With the aim of identifying the best practices for an AI-based PD assessment, as well as offering a valuable tool for pre-diagnostics and staging, we explored a broad scenario of possible solutions, considering both data-driven and model-driven approaches. We compared the reportedly most effective methodologies in feature-based ML and DL, evaluating their performances with a thorough 10-fold cross-validation procedure. Moreover, we devoted a lot of attention to the creation of a highly populated, well-stratified, and balanced dataset. Data about patients’ drug status (i.e., ON/OFF state) and impairment stage (i.e., early or mid-advanced) were also considered. In addition, an analysis of the most relevant acoustic features was carried out to confirm/deny the existing literature, allow clinical parallels and identify trends related to the level of impairment or medication.

## 2. Materials 

### 2.1. Dataset

For this study, we recruited 266 healthy control (HC) and 160 PD subjects, the latter divided into Early (72 subjects newly diagnosed) and mid-Advanced (88 subjects with medium-to-advanced impairment) patients. 

**Figure 1:**
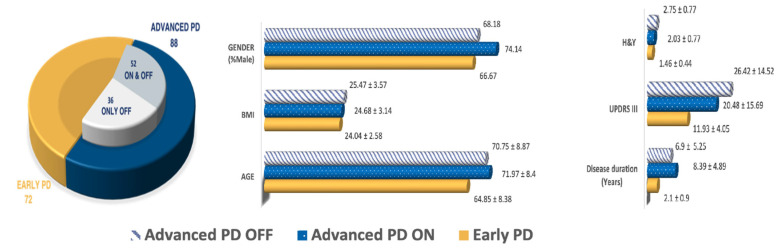
Demographics and clinical characteristics of the study population.

The diagnosis was performed by expert neurologists according to standardized diagnostic criteria [58]. Motor symptoms were scored using the H&Y and UPDRS scales; the pharmacological condition of each subject was carefully noted: 52 mid-advanced PD patients were recorded in both ON- and in OFF Levodopa (L-Dopa) state, whereas samples from the remaining subgroup were collected only in OFF state. OFF state recordings were performed at least 12 h after the last medication intake, whereas ON state recordings were performed within 1–2 h of the last administration. Early PD subjects, due to their recent diagnosis, hadn’t received any medication. Figure 1 reports a detailed description of the demographics and distribution of the PD population; HCs were selected to match the pathological subgroup in terms of age, gender, and BMI.

The selection criteria for both HCs and PDs included: (i) Italian native speakers; (ii) 18+ years old; (iii) no previous history of smoking. Subjects characterized by a respiratory, gastro-esophageal, auditory system, or vocal fold disease were also excluded.

As for the data-collection procedure, vocal samples were recorded employing either a Y6S Honor smartphone (by Huawei, Guangdong, China) or a dynamic headset microphone WH20 (by Shure, Niles, IL, USA) with XLR male 3-pin connector, together with a voice recorder H5 (by Zoom, Tokyo, Japan) in high quality and uncompressed format (.wav, 16-bit, 44.1 kHz). Smartphone recordings were collected through a dedicated application that guaranteed the absence of compression or filtering and the same sampling frequency as the professional microphones. All the samples were collected in a quiet and echo-free room.

**Figure 2:**
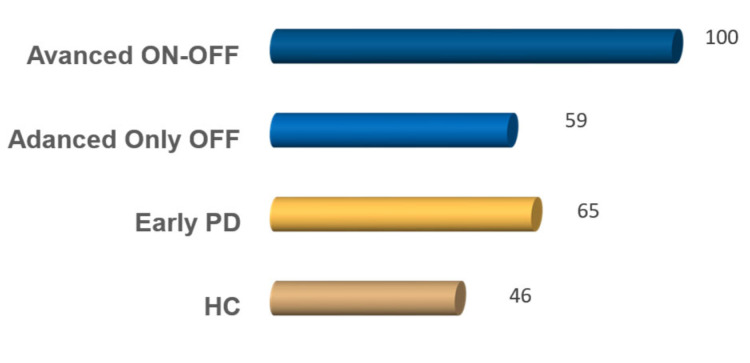
Percentage of samples for each subgroup that have been recorded with a professional microphone.

Given the possible influence of the recording modality on the VAT, we composed the dataset to maintain the same percentage of microphone and smartphone recordings for each subgroup employed for the study. The only exception to this related to the patients recorded in both ON and OFF states, which were entirely collected by means of professional equipment. Figure 2 reports more detailed information about the distribution. 

The data collection procedure involved several medical institutes, namely the Department of Human Neurosciences of the University of Rome La Sapienza, the Department of Systems Medicine of the University of Rome Tor Vergata and IRCSS Neuromed Institute Pozzilli. During each recording session, subjects were asked to sit in a relaxed position and sustain the vowel /e/ for 5 s at a comfortable volume [5]. The sustained emission of a vowel could be the most appropriate technical solution for preventing linguistic confounding and achieving standardized worldwide procedures [59]. Moreover, studies concerning neurological disease (including PD) reported that the results derived from using a sustained vowel are comparable to those obtained with connected speech [21,32,60]. To correctly perform the feature extraction procedure, we excluded signals that were too short or with low SNR. Signals processing, data analysis and model training were carried out through Python 3.8, MATLAB R2022b (MathWorks, Natick, MA, USA) and Praat6.3 [61].

Participants gave written informed consent, which was approved by the institutional ethics committee (0026508/2019), according to the Declaration of Helsinki; demographic and clinical data were noted anonymously.

### 2.2. Audio Pre-Processing

As specified above, audio data were recorded non-homogeneously using either smartphone microphones or a professional headset microphone, in both cases the file being a .wav lossless 24-bit type. The Shure WH20 headset microphone is dynamic and has a cardioid polar pattern, while smartphone microphones are MEMS-based, and omnidirectional. These characteristics do provide relatively significant differences in microphones, as cardioid patterns tend to only capture what is in front of them, and a dynamic solenoid-based technology is less sensitive and less “realistic” than a condenser or MEMS [62]. However, the silent, controlled environment in which the recordings were held and the proximity of the source (the subject’s mouth) to the microphones minimized the abovementioned differences to a certain degree.

Several studies demonstrate how relevant perceptual features do not change significantly between smartphones and comparable professional microphones, especially those related to the fundamental frequency (F0) and subsequent estimators such as Jitter, which are conversely among the most widely used and effective features in pathological voice analysis [63,64]. To further reduce any differences, we opted to have the smartphone recordings undergo a pre-processing procedure. The main differences between MEMS- and dynamic-based recordings can be summarized as the former having a higher degree of background noise due to the omnidirectional nature of the MEMS microphone, and a different frequency response. In our study, a slight degree of noise cancellation was applied using an algorithm based on spectral subtraction, individually learning the noise profile of each audio recording [65]. For the frequency response, a pre-emphasis procedure was carried out mimicking the declared response of the Shure WH20; the response of an omnidirectional MEMS microphone can, conversely, be well approximated to being flat [66]. In addition, further low-pass filtering at 12 KHz was applied to the whole dataset, since the response of smartphones decays in that region, and the amount of relevant information in voice signals is negligible. A 30-tap FIR filter implemented on MATLAB was used for this step. 

The abovementioned procedures were all successfully employed before preparing the audio data for AI, and the quality of the processed recordings as well as their perceptual similarity was evaluated empirically by a team of trained sound engineers. 

## 3. Methods 

In the next paragraphs, we will describe the two different approaches we used for the classification tasks. The first is a traditional machine learning approach, consisting in training several classification models with the most relevant selected vocal features. The second approach involves a convolutional neural network (CNN) trained on augmented Mel-spectrograms. The results obtained with the two approaches are also compared with each other with a statistical analysis, using both Student’s *t*-test [67] and Pearson’s test [68]. The actual values compared are the accuracies obtained in each fold of the cross-validation, with only the best performing algorithm being considered for each task involving ML. 

### 3.1. Traditional Machine Learning Approach

The traditional classification approach is a pipeline divided into three phases:

1. Feature extraction;
2. Feature selection;
3. Model training.

#### 3.1.1. Feature Extraction

In the first phase, we extracted 453 different vocal features from each audio recording, expressly chosen among those considered useful to assess the voice disorders caused by PD. This phase aimed to build a data matrix where each column represents a feature and each row represents a subject.

The 453 vocal features were extracted with different methods: the first group of 339 features was extracted through the Voice Analysis Toolbox [69,70,71], a MATLAB toolbox specifically designed for extracting linear and non-linear vocal features through the use of different speech signal processing algorithms; a second group of 18 features relating to low-frequency vocal tremor was extracted through Praat script *tremor.praat* v.3.05 [72,73,74]; and finally, a third group of 96 vocal formants-related features was extracted through Parselmouth, a library that provides a simple way to run Praat’s C/C++ code through Python [75], with custom routines.

The Voice Analysis Toolbox is a ready-to-use tool able to extract many of the most valuable vocal features for the quantification of dysphonia, each plausibly related to a clinical manifestation of a disease of the voice. The toolbox was used to extract features such as jitter, shimmer, HNR or MFCC and many non-linear features such as pitch period entropy or glottal-to-noise excitation [69]. Subsequently, we added some other interesting parameters that were not present among those extracted from the toolbox: the first group of added features comprise 18 vocal parameters related to the unintentional low-frequency vibration of the vocal fold, whose amplitude and frequency could be affected by the neuronal deficit caused by Parkinson’s disease [72]; the second group is composed of 96 features related to the vocal formants and their energy. Vocal formants represent the acoustic resonant frequencies of the human vocal tract, and their values depend on the position of the tongue and the characteristics of the vocal tract [76]. We extracted the first five vocal formants and then applied the Teager-Kaiser energy operator (TKEO) [77] to each of them to estimate their instantaneous energy. From each formant and its energy, we extracted 8 numerical parameters including mean, standard deviation, range, percentile and slope. A summary of the extracted features is reported in Table 2.

#### 3.1.2. Feature Selection

Some of the various dysphonia measures that were extracted could be highly correlated with each other, resulting in redundant information being transferred to the dataset. Moreover, training machine learning models through a dataset with several instances lower than the number of features could lead to poor results because of overfitting. Reducing the size of the dataset is the best solution to achieve a more efficient analysis and better classification performance. In particular, feature selection techniques can identify a small subset of the most relevant features from the original dataset, excluding the irrelevant and redundant ones [78]. In the second phase of our study, we compared three different *filter* feature selection methods, which were preferred to *wrappers* due to their independence from a specific classification model. All the feature selection procedures were performed through Python. 

The first method we considered is the classic information gain (IG) ranking method, a univariate feature selection method that ranks features in terms of their information gain with the class. IG estimates the entropy reduction due to the observation of a certain feature. Features with a high value of IG are more important because they have been able to reduce the entropy of the class:

(1) IG=H(Y)−H(Y|X)

where H(Y) represents the entropy of the class a priori, and H(Y|X) represents the conditional entropy of the class after the observation of the feature *X*. 

The second method we used is the correlation-based feature selection (CFS) algorithm, a heuristic feature selection method that seeks to identify the subset *S* that maximizes the following merit function:

(2) $$Merit_{S}=\frac{k*r_{cf}}{\sqrt{k+k\left(k-1\right)r_{ff}}}$$

where *k* is the number of features in the subset; $r_{cf}$ is the average correlation between the *k* features and the class; and $r_{ff}$ is the average inter-correlation among the *k* features [79]. 

The CFS algorithm performs a best-first kind of search [80] and estimates the correlation values through a measure called symmetrical uncertainty (SU), which compensates for the bias of the IG and normalizes the result in the range [0, 1]:

(3) $$\mathrm{SU}=2\cdot \frac{\mathrm{IG}}{H\left(Y\right)-H\left(X\right)}$$

where IG represents the information gain, while H(Y) and H(X) respectively represent the entropies of the class and of the variable. 

The third method we used is the minimum redundancy maximum relevance (mRMR), a multivariate ranking method that evaluates the features through a forward feature selection search, considering both their similarity/redundancy and relevance (i.e., correlation with the class) [81]. In our study, we used a variant of the classic mRMR algorithm introduced by Tsanas et al. and called mRMR Spearman (mRMRS) due to the use of the Spearman coefficient to evaluate the correlations [82]. The algorithm tries to find a subset composed of the features that enable the highest score to be obtained when evaluated with the following merit function:

(4) $$\mathrm{mRMR}=\underset{x\in Q-X}{\max}\left[S\left(x,y\right)-\frac{1}{n_{X}}\underset{z\in X}{\sum }S\left(x,z\right)\right]$$

where Q is the original feature set, X is the evaluated subset, $n_{X}$ is the number of features in X, and S is the Spearman correlation coefficient. The first part of the function estimates the relevance, measuring the correlation between the feature *x* and the class *y*, while the second part measures the redundancy, evaluating the correlation between two features *x* and *z*, belonging to the subset X.

#### 3.1.3. Model Training 

In the third phase, the selected features were used to train three different machine learning models: *k*-nearest neighbors (kNN), naïve Bayes (NB) and support vector machine (SVM), which were chosen because of their effectiveness in voice analysis [83]. Models were trained with a 10-fold cross-validation through the features selected by the three feature selection methods. Moreover, their performance was compared using statistical metrics such as accuracy, sensitivity, specificity and F1-score, and also through the receiver operating characteristic (ROC) curve and the area under the curve (AUC). 

In addition, since we observed that the CFS usually selects small subsets of 10 or 20 features while the other two methods are prone to assigning a non-zero score to more than 100 features, we performed an exploratory analysis of the features ranked by IG and mRMRS to find the number of the first *n* features that allowed us to obtain the best results and finally, we compared these subsets with the ones selected by CFS. 

To achieve the best performance, all the classification models went through a hyperparameter tuning using a Bayesian optimization procedure performed in MATLAB. To estimate the values of the performance-maximizing hyperparameters, the Bayesian optimization algorithm tries to minimize the misclassification function in a bounded domain without previously assuming any functional forms [84,85]. 

Of course, which hyperparameter to tune depends on the classification model considered. In particular, the optimization procedure has fine-tuned the following parameters:

- For the SVM classifier, the optimizer selected the kernel between linear or radial-basis, as well as the values of c and gamma;
- For the kNN classifier, the optimizer selected the distance/similarity metric between Euclidean, Manhattan, Chebyshev, Hamming, cosine, correlation or Mahalanobis distances;
- For the NB classifier, the optimizer performed a kernel density estimation procedure to choose the kernel function—Gaussian, triangular or Epanechnikov—and its width.

### 3.2. Deep Learning Approach

The second classification approach we used involves training a CNN through a 10-fold cross-validation using Mel-spectrograms as input images.

CNNs inherently offer high-performance analyses on image data, due to their filtering nature that allows to identify local graphical features. With DL being one of the standard solutions for audio analysis, spectrogram-based CNNs are considered the standard solution often providing state-of-the-art results.

Mel is a re-scaling of the spectrum based on discrete bands weighed according to perceptual characteristics, and is a standard representation of audio signals which reportedly offers some of the best results in voice analysis and classification [86,87]. We plotted grayscale Mel-spectrograms for all the audio recordings using a 2048 FFT and 512 points hop length. Due to the scarcity and unreliability of information within the higher frequency bands in the human voice [88], we decided to limit the frequency range to a maximum of 12 kHz. 

Since deep learning models require large training datasets to perform high quality generalization of the information, the usage of data augmentation techniques has become a well-known practice to increase the amount of training data by generating synthetic ones based on the existing training set. We employed six different audio data augmentation solutions, four of which were applied to the audio signals and two directly to the spectrograms. The techniques are reported below:

- Time stretching: slows down or speeds up the signal at a random rate between 0.6 and 1.4;
- Pitch shifting: shifts the pitch of the signal up or down by a random amount between 1 and 3 semitones;
- Noise addition: adds Gaussian noise to the original signal with an amplitude equal to 10% of the RMS value of the signal;
- Room simulation: this algorithm simulates the frequency response of a large and reverberating room;
- Time masking: covers part of the spectrogram over time with rectangular monochromatic boxes;
- Frequency masking: covers with rectangular monochromatic boxes part of the frequencies of the spectrogram.

Data augmentation was carried out only on the training folders for each iteration of the cross-validation procedure, while the validation folder contained only the original images. 

During training, the model evaluated a batch composed of 32 elements through the cross-entropy loss function via an adaptive momentum estimation (ADAM) optimizer. The elements in each batch were randomly selected from the training data, which was composed of 9 out of the 10 cross-validation folders. The model was trained for 60 epochs and its weights were saved in each epoch, to select the best model after the completion of the training; if the loss was unaltered or got worse for 25 epochs in a row, the model stopped the training early, and started evaluating the next cross-validation folder. Training optimization started with a learning rate of 0.01, which could decrease by a factor of 0.1 if the loss function got worse for 10 epochs in a row. A different validation set was used to evaluate model performance during each cross-validation epoch, and at the end, the average results were considered.

The CNN architecture was built with the aims of avoiding overfitting and maximizing performance with a good compromise in net size. Significantly, in previous works with similar tasks (voice analysis for pathology detection, especially directed towards COVID-19), we experimented with transfer learning, using “common” nets such as AlexNet or ResNet [89], but observed little to no improvement in accuracy with respect to lighter, custom-made nets [20]. We thus preferred in the current study to implement a model trained from scratch which could be the basis for future implementations of custom CNN models for the analysis of vocal tasks.

**Figure 3:**
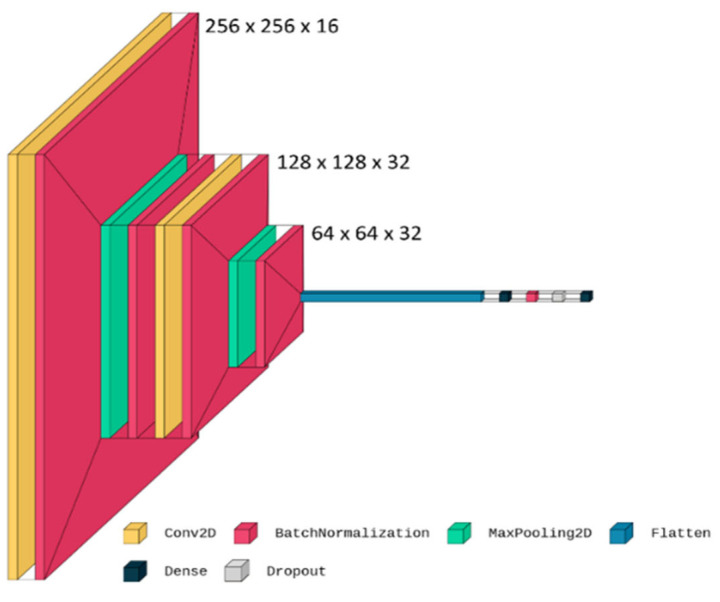
Proposed CNN architecture.

The architecture of the CNN was chosen to avoid overfitting and to maximize its performance. The proposed network receives a 256 × 256 sized image as input and is composed of two convolutional layers with 16 and 32 filters, respectively; of several batch normalization layers that follow and precede the convolutional layers; of a neural network with 32 hidden neurons; of a dropout layer with a probability of 0.5; and finally, of the output layer with softmax as activation function. A picture of the proposed architecture is presented in Figure 3. All the described procedures were performed using the Keras, Audiomentations and Librosa Python libraries [90].

**Figure 4:**
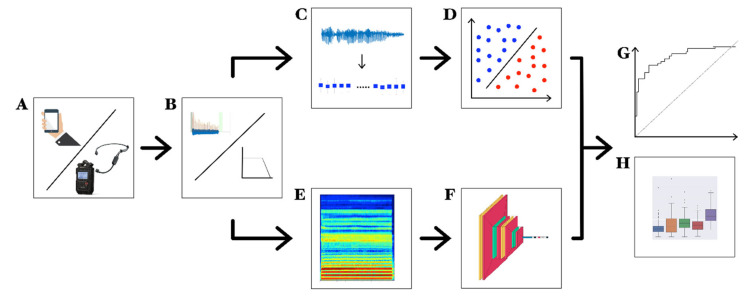
Experimental design (exemplified view): (**A**) recording of voice samples through high-definition audio recorder or smartphone; (**B**) pre-processing; (**C**) feature extraction; (**D**) ML model training (binary SVM shown as an example); (**E**) spectrogram; (**F**) CNN training; (**G**) ROC curves; (**H**) feature distributions.

A complete description of our experimental design is summarized in Figure 4.

## 4. Results

### 4.1. Traditional ML Classification Approach

In Table 3, we report the results obtained through the Bayesian hyperparameter optimization procedure applied to the three feature selection methods (i.e., CFS, IG and mRMRS). For each binary and multiclass classification and each feature selection algorithm, we reported the couple number of features–classification model that led to the best classification accuracy, which is expressed in terms of average cross-validation (CV) results. 

To further assess the effectiveness of each feature selection algorithm, Table 4 shows the classification accuracy of each method (whose internal hyperparameters were set as in Table 3) averaged over the three different ML models tested (i.e., KNN, NB, and SVM). Results are expressed in terms of CV accuracy.

In a specular way, to evaluate the robustness of each classification model, in Table 5 we report the performance yielded by each tested ML model averaged across the three feature selection models employed for the study. In this case, results are expressed in terms of CV classification accuracy. 

**Figure 5:**
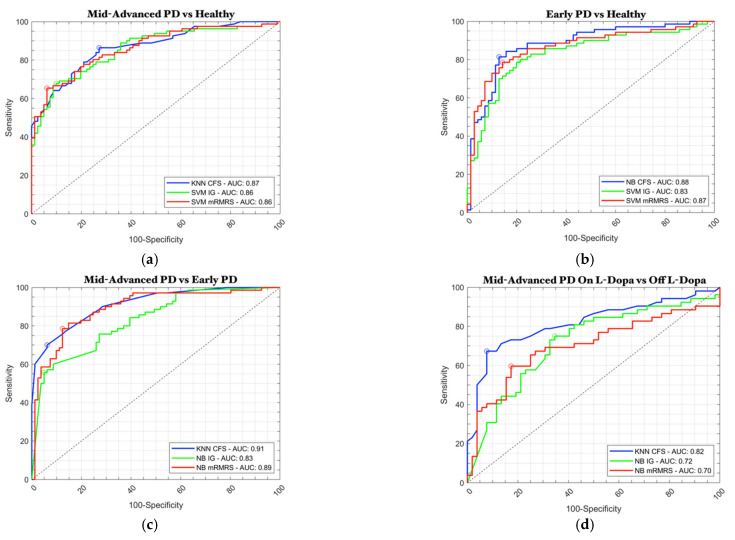
ROC curve comparison: (**a**) mid-advanced-stage PD patients vs. healthy control group; (**b**) early-stage PD patients vs. healthy control group; (**c**) mid-advanced-stage vs. early-stage PD patients; (**d**) mid-advanced-stage PD patients ON L-Dopa therapy vs. OFF L-Dopa therapy.

In Figure 5, we show the ROC curves and their relative area under the curve (AUC) for each binary classification task, showing all three feature selection methods with the classification models that perform best, as reported in Table 3. Since the performances of the three classifiers are comparable, we decided to graphically compare the three feature selection methods to enable their differences to be more readily visualized.

### 4.2. Comparison between Classic ML and CNN Models

**Figure 6:**
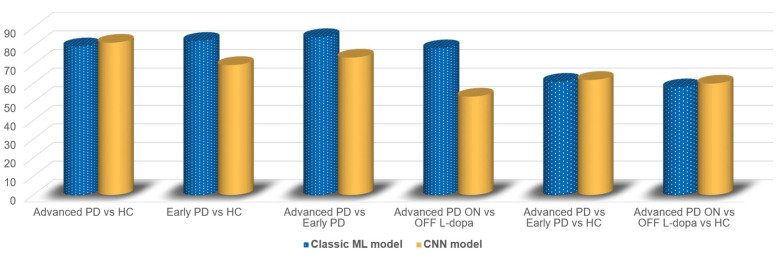
Comparison between the best classic ML model fed with handcrafted features and a CNN model fed with spectrogram images. Results are expressed in terms of CV classification accuracy. L-Dopa therapy ON vs. OFF.

Figure 6 depicts a comparison between classic ML and CNN models, both of whose performance are expressed in terms of CV classification accuracy. In the case of the classic ML model, we report the combination of feature selection classification models which led to the best performance according to the previously exposed results (see Table 3, Table 4 and Table 5). 

For the sake of completeness and to enhance the comparison of the proposed approach with similar studies, in Table 6 and Table 7, we report the complete set of metrics (i.e., accuracy, positive predictive value, negative predictive value, sensitivity, specificity, area under the curve, and F1 score) used to assess and compare the performance of each algorithm, for binary and multiclass classifications, respectively. The statistical analyses were carried out on all the binary tasks bar mid-advanced PD ON vs. OFF L-dopa, in which the differences between ML (KNN) and CNN are too skewed, and may bias the statistics when the aim is to identify possible unwanted correlations. The results of the *t*-test show a two-tailed *p*-value of 0.0346 (t = −2.5403), while Pearson’s test reveals an r value of 0.034. By common standards, both tests convey metrics associated with little to no statistically significant correlation between the variables [68]. 

### 4.3. Vocal Biomarkers

Table 8 reports the feature selection results for each binary and multiclass classification carried out in the current study. For the sake of brevity, we reported the 5 top-ranked parameters for each feature selection method. 

**Figure 7:**
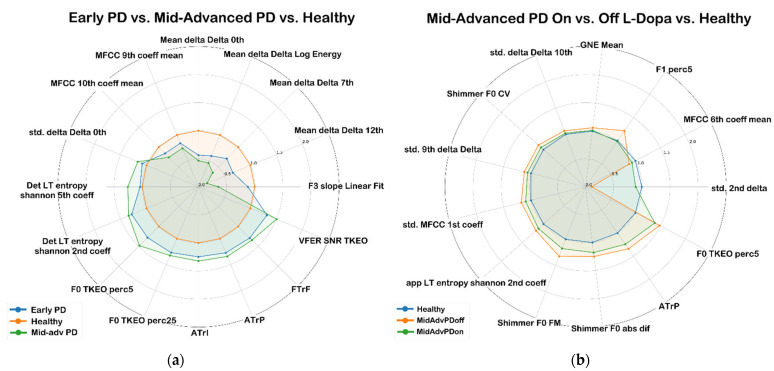
Spider plots of some of the most significant features for different comparison scenarios: (**a**) most significant features in differentiating between early or mid-advanced PD subjects and HCs; (**b**) most significant features in differentiating between ON and OFF L-Dopa mid-advanced PD subjects.

To derive information regarding the effectiveness of the selected features, in Figure 4, we report two spider plots with the most significant features for (i) early identification of PD disease; (ii) evaluating the effect of the medication; (iii) monitoring the progression of the disease. Features represented in Figure 7 were chosen among all those in the highest positions of the ranking after being individually analyzed to find those that allow a better separation of the distributions of the classes. For each feature, we report the average values normalized over the whole HC population to highlight eventual differences between normophonic and non-normophonic voices.

## 5. Discussion

Results show that traditional machine learning approaches can discriminate with high accuracy between the voices of the patients affected by Parkinson’s disease and of healthy controls, even if the patients are only in the early stage of the disease. Moreover, it is possible to distinguish the voices of the mid-advanced stage patients before and after the therapy. The accuracies of the multiclass classifications are not as good as the ones of the binary classifications, but the results obtained are in line with the expectations.

The average accuracy reached for the binary classifications which involve all the possible PD states considered in this study (healthy, early, mid-advanced ON and OFF L-Dopa) is 82.25%, calculated on the basis of the best performing models. However, taking a closer look at Table 6 and Table 7, it can be seen that “traditional” ML models (KNN or SVM) provide a higher performance three times out of four, with a mean accuracy of 81.75% versus 69.75% reached by the CNN–albeit affected by the very low 53% reported for the mid-advanced ON vs. OFF comparison. Moreover, a slight advantage for CNNs is reported for multiclass tasks, with a 61% mean accuracy versus the 59.5% reported by the traditional ML methods for the three classes. The higher generalization power of ML models is also confirmed by the area under the ROC curves being consistently higher, with a mean of 0.87. 

Overall, these results show how traditional ML methodologies still hold a relevant place for highly complex tasks such as voice analysis with low-cardinality datasets; on a side note, as limited as the study population might be, this remains a work involving one the biggest datasets for PD detection to-date [91]. Thus, as many studies and results such as [20,92] and [93] suggest, ML algorithms can still provide significant results, sometimes improving the state-of-the-art diagnosis, if carefully fine-tuned and applied to the correct features.

As shown in Table 5, the most effective algorithm for the present tasks appears to be the KNN, which conversely is also one of the simplest. However, the widespread SVM does provide comparable performance, and even has a slight edge for multiclass tasks.

Furthermore, a much bigger difference is observed when comparing different feature selection methods (Table 4). The CFS appears to consistently provide the best performance over IG and mRMRS, with the latter coming in second place. We remark that CFS involves a search method, which was Best-First in our case [80], and thus retains a non-standardized number of features depending on the task; conversely, information gain or mRMRS are used as rankers and do not provide the performance of CFS even when using the empirical best number of features. 

CFS and mRMRS can be roughly based on the same principle of valuing the high correlation of a feature with the class and devaluing inter-correlation between features. With these premises, the main differences between the two methodologies could be summarized as the statistical indicators used to compute each correlation, and the different search methods that we employed—a ranker implying a single-feature-wise search dynamic. By contrast, information gain is based on the amount of information gained about a random variable (class) from observing another random variable (feature), and does not include considerations about the inter-correlation between features, thus creating the risk of redundant sets. IG constantly yields the lowest performance. 

Hence, the observations within this study point to ML methods still achieving results that are comparable to, if not better, than those from deep learning in experimental environments such as those of vocal analysis, often involving reduced datasets and complex tasks, as confirmed by the statistical analyses. ML algorithms also are proven to be more reliable, offering more consistent results. We would like to stress that most of our previous work within the same context point to the same conclusion, even when using transfer learning and comparing architectures [20,24]. Moreover, the plethora of studies involving voice analysis for PD, albeit showing a trend towards the usage of CNNs in the last few years, still achieve equally relevant results with ML methods [52]. However, let the reader be reminded that accuracies and trends in specific tasks with limited datasets can only point out a “direction” for future studies to take, define a more thorough baseline methodology and present all the possible viable alternatives, and it is not recommended to draw strict conclusions on the accuracy of specific models. 

A delicate choice of algorithms and a thorough tuning procedure are necessary to build the best-performing ML pipeline, with CFS being identified as the most effective selector among those here analyzed, and SVM and KNN being the most effective algorithms. The advantage of a CNN-based approach is clearly in the “black box”-like behavior that does not require such attention to detail or the choice of features and extraction methods; nevertheless, differences in net architecture, optimization algorithm and data preprocessing and augmentation remain relevant. 

Taking a closer look at Table 1 and in general on the overview of the current state-of-the-art of voice analysis for PD (as detailed in [52]), we believe that the strengths of this work can be summarized as: the extensive set of well-recorded, validated data; a comprehensive approach comparing ML and CNN methodologies; and the usage of a broad spectrum of acoustic features. 

We extracted a set of 453 features, chosen from all those that previous researchers considered relevant for quantifying voice disorders caused by PD. We strove to present a single corpus that included all the parameters deemed interesting by the various studies that exist in the literature. Our feature set includes several linear and non-linear measures extracted through the voice analysis toolbox, such as prosodic and spectral features, MFCC, wavelet decomposition or GNE, to which we added features related to low-frequency vocal tremor and the vocal formants. In this work, we have analyzed how the distributions of these values vary with the progression of the disease and with the L-Dopa therapy to find clinical biomarkers useful for an early diagnosis of PD and the evaluation of the medical therapy. 

As far as acoustic features are concerned, F0, shimmer and jitter could be summarized as the most widely used ones in vocal analysis for PD. Looking at the exemplified overview of the top-ranked features in Table 8, the results of CFS and mRMRS are quite similar, which confirms the fact that they are based on the same dynamics. The lesser-performing IG usually detects different features as the most relevant. Quality-wise, most of the features identified by CFS/mRMRS are indeed related to perceptual characteristics such as F0, shimmer, formants (e.g., F1, F5) or glottal model-based macroscopic indicators (e.g., “VFER”: vocal fold excitation ratio) with only a partial amount being composed of high-level differential features (e.g., “std_10thDelta” which identifies the tenth Delta coefficient of the standard deviation of the windowed signal). Conversely, IG often appears to rely on such features, which provide a high level of abstraction and are very difficult to interpret from a perceptual point of view. Paired with the consideration that CFS appears as the best-performing feature selector, the trends in the top-ranked features preliminarily confirm how pitch-related and prosodic features bear relevant information for the detection and staging of PD in voice.

## 6. Conclusions and Future Work

Due to the empirical nature of the current methodologies for diagnosing PD, and the ongoing experimentations regarding treatment and dosages, it is crucial to build reliable support, and investigate the promising characteristics of voice analysis. Many solutions have been proposed in the literature, which rely on several different ML or DL algorithms, and use baseline datasets that all share the characteristics of low cardinality. With these premises, we strove to build as wide and carefully recorded as possible a dataset, providing 160 PD patients and 266 healthy controls which, albeit still small, stands out as one of the largest PD datasets so far. To assess the robustness of the proposed classic ML approaches, we compared several pipelines of feature selection classification algorithms and investigated the performance variability introduced when varying each block composing the pipeline. According to our findings, changing the feature selection method has the highest impact on the classification accuracy, as demonstrated in Table 4. As for the specific algorithm, CFS was revealed to be the most effective one, leading to an increase of around 5% compared to the mRMRS and IG. Moreover, CFS typically returns several selected features in the range of 10–20, which enhances rapid model training and reduces the possibility of overfitting. Results also show how ML can still achieve results comparable to CNN, with the added advantage of being more reliable in terms of accuracy, and highly interpretability due to it being based on acoustic features. CFS is proven to be the best feature selector among those analyzed, and SVM and KNN provide similarly good classification performance.

CNN often provides similar results to ML, with the only exception being the task of differentiating mid-advanced PD patients ON and OFF L-Dopa–which conversely is the only differential analysis here presented. CNNs also do not require such a detailed fine-tuning of features, internal algorithms and hyperparameters.

Binary classifications among all classes (healthy, early, mid-advanced ON and OFF L-Dopa) resulted in a maximum mean accuracy of 82.25, while three-class tasks only peaked at 61%. A mean AUC of 0.87 confirms the generalization power of the proposed algorithms. 

Even within classical clinical environments, identifying consistent thresholds for PD stages is a difficult task, often relying on partially-exhaustive indicators such as UPDRS. The post-hoc analysis conducted on the selected features showed MFCC and their derivatives to be the most frequently selected feature (Table 7), especially for mid-advanced PD patients, thus suggesting their ability to describe the disease progression. A significant number of features associated with F0, shimmer, and jitter, which are a common standard in voice analysis, are selected when comparing vocal samples from ON and OFF L-Dopa PD patients, thus confirming previous evidence from [28,29]. 

Finally, wavelet decomposition measures, low-frequency tremor features and glottal-to-noise excitation (GNE) demonstrated higher effectiveness in discriminating early stages of the disease from the healthy control group or the mid-advanced-stage PD. 

Despite the promising results, we also acknowledge the presence of several limitations that still must be addressed. Regardless of whether the size of the employed datasets is statistically significant and generally higher than in similar studies, we plan to further increase the sample and validate our findings on larger datasets, to obtain better and more reliable results, especially with CNN. As for the protocol used in our study, we employed a single speech task (that is, sustained vowel phonation), which on the one hand enables language-independent results, but on the other can lead to suboptimal results. Future studies will take into account additional analyses to investigate possible improvements due to a more complete set of tasks performed. The limitations of our study can be identified in a dataset that, albeit bigger than the vast majority of other datasets in the field, still cannot be compared to the extensive, big data-like sets commonly employed in other successful AI tasks with high generalization. As far as algorithms are concerned, a more thorough experimentation of CNN techniques and architectures could be useful, although transfer learning has proven to give no relevant advantages in our past studies. With the aim of identifying the benefits given by each processing step, we also will try to implement an ablation analysis for the data augmentation procedures in our future research. Moreover, although we carefully pre-processed our dataset to mitigate the presence of different recording equipment, we are aware that this could have negatively affected the performance. However, this reflects the condition of a real-world scenario to which we aspire: the development of an automatic tool to monitor and evaluate the progression of PD which is independent of external conditions.

## Figures and Tables

**Table 1:** Brief literature overview (more in the literature review by Amato et al. [52]). All references to datasets and methodologies can be found in the corresponding paper. Accuracies are averaged if not specified.

Study	Dataset	Classification Approach	Reported Results (ACC)	Notes and Limitations
Jeancolas et al., 2022 [53]	256 (117 PD)	SVM	79.5%	Also takes into account RBD patients (ACC = 63%). The features extracted are not detailed and in general, it is too little a subset.
Hireš et al., 2022 [54]	100 (50 PD)	CNN	99% (vowel /a/)	Small dataset (PC-GITA). Only vowel tasks are considered, with /a/ being reported as the most effective.
Er et al., 2021 [55]	100 (50 PD)	CNN and LSTM	98.5%	Small dataset (PC-GITA). Several pre-trained nets are employed, especially ResNet variants.
Govindu et al., 2023 [56]	149 (100 PD)	SVM, linear regression, Random Forest, KNN	91.8% (Random Forest)	Small, unbalanced dataset consisting of just a few speech features (no audio). Upsampling was used to address imbalance and wrangling was used to infer missing attributes.
Carrón et al., 2021 [57]	UEX (60 total, 30 PD) and mPower (1060 PD)	Gradient Boosting, Logistic Regression, Passive Aggressive, MLP, Random Forest, SVM	92% (UEX), 71% (m-Power)	The mPower dataset is crowdsourced, non-validated and self-reported. On the other hand, the proposed UEX dataset is very small (30 PD). Only 33 features are used, including the sex of the subject.

**Table 2:** Summary of the 453 extracted features.

Feature Family	Number of Features	Brief Description	ID
Fundamental Frequency	2	Lowest frequency of the quasi-periodic vocal signal, which represents the vibration frequency of the vocal folds	F0
Jitter	22	Variability/perturbation of the fundamental frequency	Jitter
Shimmer	22	Voice amplitude perturbation	Shimmer
HNR/NHR	4	Harmonic-to-noise ratio	HNR, NHR
Mel Frequency Cepstral Coefficients	82	Cepstral coefficients that estimate the filtering effects of the vocal tract on the sustained emission	MFCC
Vocal Formants	96	Vocal tract resonance frequencies, which are related to tongue position and vocal tract morphology	F1, F2, F3, F4, F5
Detrended Fluctuation Analysis	1	An estimate of the turbulent air-flow that traverses the vocal tract	DFA
Recurrence Period Density Entropy	1	This measures the stability of the oscillation produced by the vocal folds evaluating the periodicity of the signal	RPDE
Pitch Period Entropy	1	This evaluates the stability of the intonation (pitch) during the emission of a sustained vowel without being confused by the microtremor present even in healthy voices	PPE
Wavelet Decomposition Measures	182	Signal decomposition through the discrete wavelet transform (DWT) for the purposes of calculating the energy present in the various frequency sub-bands	WavDec_det (detailed coefficient) WavDec_app (approximate coefficient)
Empirical Mode Decomposition Excitation Ratio (EMD-ER)	6	This decomposes the signal through the intrinsic mode functions (IMF) and analyzes them to quantify the noise due to an incomplete glottal closure through entropy and SNR measurements	IMF
Glottis Quotient	3	A measure of the aperiodicity of the glottal cycle	GQ
Glottal-to-Noise Excitation Ratio	6	An estimate of the noise caused by the incomplete closure of the vocal folds calculated by cross-correlating the envelopes of the glottal cycles	GNE
Vocal Fold Excitation Ratio	7	This estimates noise unrelated to the vocal emission, similarly to GNE	VFER
Low-Frequency Vocal Tremor	18	Parameters related to the unintentional low-frequency oscillations of the vocal fold and their amplitude	Trem

**Table 3:** Comparison across the three feature selection algorithms employed. For each method, we report the couple number of features–ML model that enhances the best performance, and the corresponding classification accuracy. Results are expressed as k-fold CV average and standard deviation.

Comparison	Selection Method	Number of Features	Classification Model	Accuracy
Mid-Advanced PD vs. HC	CFS	12	KNN	0.80 ± 0.008
	IG	100	SVM	0.74 ± 0.04
	mRMRS	50	SVM	0.77 ± 0.008
2. Early PD vs. HC	CFS	17	NB	0.82 ± 0.007
	IG	30	SVM	0.78 ± 0.16
	mRMRS	70	SVM	0.83 ± 0.02
3. Mid-Advanced PD vs. Early PD	CFS	17	KNN	0.85 ± 0.02
	IG	30	NB	0.79 ± 0.02
	mRMRS	10	NB	0.78 ± 0.01
4. Mid-Advanced PD ON vs. OFF L-dopa	CFS	10	KNN	0.79 ± 0.005
	IG	10	NB	0.66 ± 0.03
	mRMRS	10	NB	0.69 ± 0.016
5. Mid-Advanced PD vs. Early PD vs. HC	CFS	21	KNN	0.61 ± 0.03
	IG	70	KNN	0.60 ± 0.01
	mRMRS	10	SVM	0.60 ± 0.01
6. Mid-Advanced PD ON vs. OFF L-dopa vs. HC	CFS	21	NB	0.58 ± 0.01
	IG	100	SVM	0.54 ± 0.04
	mRMRS	70	KNN	0.54 ± 0.03

**Table 4:** Classification accuracy with respect to each feature selection algorithm employed. The results are expressed as the average (and standard deviation) of the CV performance yielded from KNN, NB, and SVM models.

	Comparison	CFS	IG	mRMRS
Binary Classifications	1. Mid-Advanced PD vs. HC	0.78 ± 0.09	0.73 ± 0.04	0.75 ± 0.05
	2. Early PD vs. HC	0.80 ± 0.05	0.74 ± 0.02	0.78 ± 0.04
	3. Mid-Advanced PD vs. Early PD	0.84 ± 0.01	0.75 ± 0.02	0.75 ± 0.02
	4. Mid-Advanced PD ON vs. OFF L-dopa	0.72 ± 0.05	0.56 ± 0.1	0.63 ± 0.06
	Average	0.78 ± 0.05	0.70 ± 0.09	0.73 ± 0.07
Multiclass Classifications	5. MID-Advanced PD vs. Early PD vs. HC	0.61 ± 0.01	0.57 ± 0.02	0.59 ± 0.02
	6. Mid-Advanced PD ON vs. OFF L-dopa vs. HC	0.55 ± 0.03	0.50 ± 0.04	0.50 ± 0.03
	Average	0.57 ± 0.05	0.54 ± 0.05	0.55 ± 0.06

**Table 5:** Classification accuracy with respect to each ML model employed. The results are expressed as the average (and standard deviation) of the CV performance yielded by CFS, IG, and mRMRS.

	Comparison	KNN	SVM	NB
Binary Classifications	1. Mid-Advanced PD vs. HC	0.75 ± 0.04	0.75 ± 0.02	0.74 ± 0.03
	2. Early PD vs. HC	0.79 ± 0.04	0.79 ± 0.04	0.78 ± 0.04
	3. Mid-Advanced PD vs. Early PD	0.79 ± 0.05	0.79 ± 0.05	0.78 ± 0.03
	4. Mid-Advanced PD ON vs. OFF L-dopa	0.69 ± 0.08	0.66 ± 0.08	0.67 ± 0.07
	Average	0.76 ± 0.04	0.75 ± 0.06	0.74 ± 0.05
Multiclass Classifications	5. Mid-Advanced PD vs. Early PD vs. HC	0.59 ± 0.03	0.60 ± 0.03	0.59 ± 0.02
	6. Mid-Advanced PD ON vs. OFF L-dopa vs. HC	0.53 ± 0.03	0.51 ± 0.02	0.54 ± 0.03
	Average	0.56 ± 0.04	0.57 ± 0.05	0.56 ± 0.03

**Table 6:** Binary classification performance of the best traditional ML and CNN models. Results are reported in terms of CV accuracy. Acc: accuracy; PPV: positive predictive value; NPV: negative predictive value; Sen: sensitivity; Spec: specificity; AUC: area under the curve.

Comparison	Model	Acc	PPV	NPV	Sen	Spec	AUC	F1 Score
1. Mid-Advanced PD vs. HC	KNN	0.80 ± 0.01	0.79 ± 0.03	0.80 ± 0.02	0.80 ± 0.01	0.79 ± 0.02	0.87 ± 0.04	0.80 ± 0.03
	CNN	0.82 ± 0.07	0.87 ± 0.05	0.78 ± 0.06	0.75 ± 0.04	0.87 ± 0.05	0.83 ± 0.05	0.79 ± 0.05
2. Early PD vs. HC	SVM	0.83 ± 0.02	0.81 ± 0.01	0.83 ± 0.01	0.83 ± 0.03	0.82 ± 0.02	0.88 ± 0.05	0.82 ± 0.01
	CNN	0.70 ± 0.06	0.72 ± 0.04	0.75 ± 0.03	0.73 ± 0.04	0.66 ± 0.07	0.73 ± 0.02	0.70 ± 0.03
3. Mid-Advanced PD vs. Early PD	KNN	0.85 ± 0.02	0.77 ± 0.05	0.86 ± 0.02	0.83 ± 0.02	0.81 ± 0.03	0.91 ± 0.06	0.80 ± 0.04
	CNN	0.74 ± 0.09	0.75 ± 0.05	0.76 ± 0.06	0.69 ± 0.08	0.75 ± 0.07	0.75 ± 0.05	0.68 ± 0.05
4. Mid-Advanced PD ON vs. OFF L-dopa	KNN	0.79 ± 0.01	0.71 ± 0.02	0.87 ± 0.05	0.84 ± 0.01	0.75 ± 0.02	0.82 ± 0.03	0.77 ± 0.03
	CNN	0.53 ± 0.08	0.53 ± 0.06	0.57 ± 0.08	0.69 ± 0.05	0.37 ± 0.08	0.58 ± 0.05	0.65 ± 0.06

**Table 7:** Multiclass classification performance of the best traditional ML and CNN models. Results are reported in terms of CV accuracy. Acc: accuracy; PPV: positive predictive value; NPV: negative predictive value; Sen: sensitivity; Spec: specificity; AUC: area under the curve.

Comparison	Model	Macro-Acc	Macro-PPV	Macro-Sen	Macro-F1 Score
5. Mid-Advanced PD vs. Early PD vs. HC	KNN	0.61 ± 0.03	0.61 ± 0.02	0.61 ± 0.03	0.60 ± 0.03
	CNN	0.62 ± 0.03	0.58 ± 0.03	0.57 ± 0.04	0.56 ± 0.03
6. Mid-Advanced PD ON vs. OFF L-dopa vs. HC	NB	0.58 ± 0.01	0.56 ± 0.01	0.57 ± 0.02	0.54 ± 0.01
	CNN	0.60 ± 0.03	0.58 ± 0.04	0.49 ± 0.05	0.53 ± 0.04

**Table 8:** Best five features according to the three different feature selection algorithms employed. Results are reported for each binary and multiclass analysis performed.

	1. Mid-Advanced PD vs. HC		
Rank	CFS	mRMRS	IG
1	MFCC_std_8thDelta_delta	MFCC_std_8thDelta_delta	MFCC_std_10thDelta_delta
2	MFCC_std_11thDelta	WavDec_det_TKEO_mean_1_coef	MFCC_mean_5thDelta_delta
3	MFCC_std_1stCoef	MFCC_mean_deltaDeltaLogEnergy	MFCC_std_8thDelta_delta
4	VFER_SNR_TKEO	Shimmer__F0_abs_dif	MFCC_std_8thDelta
5	MFCC_std_10thCoef	GNE__std	MFCC_mean_6thDelta
	2.Early PD vs. HC		
Rank	CFS	mRMRS	IG
1	MFCC_std_4thDelta	WavDec_app_entropy_log_2_coef	Trem_ATrPS
2	WavDec_app_LT_entropy_log_9_coef	MFCC_mean__4thCoef	WavDec_det_Ed2_1_coef
3	IMF_NSR_entropy	WavDec_det_LT_TKEO_mean_3_coef	WavDec_app_entropy_log_6_coef
4	Trem_FTrCIP	WavDec_det_entropy_shannon_1_coef	WavDec_app_LT_entropy_shannon_1_coef
5	MFCC_std_1stDeltaDelta	MFCC_std_3rdCoef	WavDec_det_LT_entropy_shannon_1_coef
	3.Mid-Advanced PD vs. Early PD		
Rank	CFS	mRMRS	IG
1	MFCC_std_10thDelta	MFCC_std_10thDelta	MFCC_std_8thDelta
2	MFCC_std_10thDelta_delta	MFCC_mean_7thDelta_delta	MFCC_std_10thDelta
3	MFCC_std__10thCoef	GNE__std	MFCC_std_10thDelta_delta
4	GNE__std	Shimmer__F0_PQ3_generalised_Schoentgen	WavDec_app_LT_TKEO_mean_3_coef
5	MFCC_std__7thCoef	F0_slopeLinFit	MFCC_std_9thDelta
	4.Mid-Advanced PD On vs. Off L-dopa		
Rank	CFS	mRMRS	IG
1	Jitter__F0_PQ5_classical_Baken	MFCC_mean__6thCoef	WavDec_app_LT_TKEO_std_6_coef
2	F1_TKEO_mean	F0_slopeLinFit	Trem_AMoN
3	F5_rangePerc	F5_TKEO_perc95	MFCC_std_11thDelta
4	WavDec_det_LT_entropy_shannon_2_coef	MFCC_std_2ndDelta	F4_perc5
5	mean_MFCC_6thCoef	F1_perc5	Jitter__F0_PQ11_classical_Schoentgen
	5.Mid-Advanced PD vs. Early PD vs. HC		
Rank	CFS	mRMRS	IG
1	MFCC_std_10thDelta_delta	MFCC_std_10thDelta	MFCC_std_10thDelta_delta
2	MFCC_std_10thDelta	MFCC_mean_7thDelta_delta	MFCC_std_8thDelta
3	GNE__std	F3__TKEO_slopeLinFit	MFCC_std_3rdDelta
4	WavDec_app_LT_TKEO_mean_3_coef	Shimmer__F0_PQ3_generalised_Schoentgen	MFCC_std_8thDelta_delta
5	Shimmer__F0_PQ3_generaised_Schoentgen	GNE__std	WavDec_app_LT_entropy_log_7_coef
	6. Mid-Advanced PD On vs. Off L-dopa vs. HC		
Rank	CFS	mRMRS	IG
1	Shimmer__F0_DB	Shimmer__F0_PQ11_classical_Schoentgen	MFCC_std_8thDelta_delta
2	Shimmer__F0_PQ5_classical_Schoentgen	MFCC_mean_2ndDelta_delta	MFCC_std_11thDelta
3	F0__TKEO_perc25	Shimmer__F0_PQ11_classical_Baken	MFCC_std_10thDelta_delta
4	Shimmer__F0_abs_dif	Jitter__F0_TKEO_prc25	WavDec_det_TKEO_std_1_coef
5	Shimmer__F0_TKEO_prc75	Shimmer__F0_TKEO_prc75	MFCC_std_8thDelta

## Data Availability

The data presented in this study are available upon reasonable request from the corresponding author. The data are not publicly available due to privacy reasons.
